# T98. THE EFFECT OF SUBSTANCE USE ON 10-YEAR OUTCOME IN FIRST EPISODE PSYCHOSIS – EARLY CESSATION RESULTS IN BETTER OUTCOMES

**DOI:** 10.1093/schbul/sby016.374

**Published:** 2018-04-01

**Authors:** Melissa Weibell, Ingrid Melle, Inge Joa, Jan Olav Johannessen, Robert Jørgensen, Wenche ten Velden Hegelstad

**Affiliations:** 1 Stavanger University Hospital; 2 University of Oslo

## Abstract

**Background:**

The pre-TIPS study in 1994–95 showed that the duration of untreated psychosis (DUP) was long in our region with a mean value of 2.1 years, and median 26 weeks. This set the stage for the TIPS-study (1997–2000), reducing DUP through information campaigns targeted to the general population and other referral agents (GP’s, schools and others) in Rogaland County (Norway). The information campaigns were launched together with a low threshold organization with direct access to an early detection team, for a diagnostic interview and help. No referral other than a phone call needed, and a guarantee of assessment within 24 hours if there was suspicion of psychotic symptoms. The hypothesis was that this could change help seeking behavior, awareness towards psychosis and thus reduce the DUP. The information campaigns and the early detection team were introduced in an early detection(ED) area (Rogaland county, Norway) comparing DUP with two usual-detection control sites in Oslo (Norway) and Roskilde (Denmark). As a result, DUP in the early detection area was reduced from 26 weeks median to 4 weeks median.

All patients with First Episode Psychosis in the early detection area have been followed for ten years, and a twenty-year follow-up is to take place shortly. Symptom and function advantages of early detection and DUP reduction have been demonstrated as being significant throughout the follow-up period. Social and functional outcome have been increasingly emphasized as being key parameters, as these contribute to both quality of life and to financial costs in society.

Substance use is common in first-episode psychosis (FEP) and has been linked to poorer outcomes with more severe psychopathology and higher relapse rates. Early substance discontinuation appears to improve symptoms and function. However, studies vary widely in their methodology, and few have examined patients longitudinally, making it difficult to draw conclusions for practice and treatment.

**Methods:**

We aimed to investigate the relationship between substance use and early abstinence and the long-term course of illness in a representative sample of FEP patients.

Out of 301 included patients, 266 could be divided into four groups based on substance use patterns during the first two years of treatment: persistent users, episodic users, stop-users and non-users. Differences in clinical and functional during the follow-up period were assessed using linear mixed effects (lme) models for the analysis of repeated measures data.

**Results:**

Patients who stopped using substances within the first two years after diagnosis had outcomes similar to those who had never used with less symptoms than episodic or persistent users. Both episodic and persistent users had lower rates of symptom remission than non-users, and persistent users also had more negative symptoms than those who stopped using.

**Discussion:**

Our findings emerge from one of very few long-term longitudinal studies examining substance use cessation in FEP with 10-year follow-up. The results convey hope that the detrimental effects of substance abuse on mental health may be significantly reversed if one stops the abuse in time. This can help patients who struggle with addiction with their motivation to embrace abstinence.

